# Effect of Bi Addition on the Heat Resistance of As-Extruded AZ31 Magnesium Alloy

**DOI:** 10.3390/ma16030996

**Published:** 2023-01-21

**Authors:** Qinghang Wang, Haowei Zhai, Li Wang, Lixin Huang, Jun Zhao, Yuyang Gao, Bin Jiang

**Affiliations:** 1 College of Materials Science and Engineering, Chongqing University, Chongqing 400044, China; 2 School of Mechanical Engineering, Yangzhou University, Yangzhou 225127, China; 3 CITIC Dicastal Co., Ltd., Qinhuangdao 066000, China; 4 School of Mechanical and Electrical Engineering, Hunan City University, Yiyang 413002, China

**Keywords:** Mg-Al-Zn-Bi alloy, extrusion, dynamic precipitation, hot-compression, heat resistance

## Abstract

In this work, we investigate the impact of Bi addition on the heat resistance of as-extruded AZ31 alloy during high-temperature annealing and hot compression. Electron backscattered diffraction (EBSD) technique and quasi in situ scanning electron microscopy (SEM) are used to analyze the evolution of microstructures during high-temperature annealing and hot compression, respectively. The test results show that with a prolonged annealing time, the as-extruded AZB313 alloy exhibited a lower grain growth rate, due to the pinning effect of Mg_3_Bi_2_ phases distributed at grain boundaries. On the other hand, as the compressive temperature increased, the downtrend of strength is delayed in the as-extruded AZB313 alloy. Thermally stable Mg_3_Bi_2_ phases dispersed within the grains act as barriers, hindering the motion of dislocations, which not only provides a more effective precipitation strengthening effect, but also increases the resistance to deformation of grains. Moreover, grain boundary sliding can also be restricted by Mg_3_Bi_2_ phases located at grain boundaries. This work provides a new idea for the development of heat-resistant wrought Mg alloys.

## 1. Introduction

Saving energy and protecting the environment are important issues related to sustainable development. Based on global energy conservation and emission reduction policies, it is necessary to vigorously develop lightweight technology, especially for the use of lightweight materials, which has very important strategic significance. As candidates, magnesium (Mg) and its alloys show several excellent characteristics, including low density, high specific strength/stiffness, and good vibration reduction performance [1]. At present, among the different types of Mg alloys, the AZ series (AZ31, AZ80, and AZ91) is one of the most widely used due to the excellent casting properties of these alloys. However, once the operating temperature exceeds 398 K, the mechanical properties of these alloys tend to deteriorate [2]. Therefore, improving the heat resistance of AZ series alloys is an urgent task and meaningful for enlarging the application range of Mg products.

In order to achieve the stated purpose, many researchers have attempted to add alloying elements to the AZ series alloys, and this has been considered as one of the most effective methods [3,4,5,6,7]. For example, recently Li et al. [3] investigated the effect of Ce addition on the hot deformation behavior and microstructure evolution of as-cast AZ80 alloy. They pointed out that the formation of Al_4_Ce phases with a high melting point was beneficial for impeding the movement of dislocations and inducing higher peak stress in as-cast AZ80-Ce alloy than in as-cast AZ80 during hot compression. Similarly, adding Sn to the as-cast AZ80 alloy was also found to have a positive effect on enhancing its high-temperature compressive strength by forming thermally stable Mg_2_Sn phases and suppressing the dynamic precipitation of softening phases (Mg_17_Al_12_) [4]. Besides the mentioned elements, as-cast Ca/Sr/Y-containing AZ series alloys have also demonstrated that they can provide excellent heat resistance [5,6,7]. To enrich the low-cost high-performance AZ series alloys, seeking new alloying elements for addition has become a research hotspot.

In recent years, researchers have focused on the element Bi, owing to the abundance of Bi reserves and its nontoxicity to humans. In Mg alloys with Bi as the main added element, Mg_3_Bi_2_ phase is easy to form, guaranteeing the microstructural stability of the alloys at high temperature. As reported by Jin et al. [8] and Go et al. [9], Mg-5Bi-3Al (BA53, in wt%) alloy could be successfully extruded without any surface cracking at 673 K and at high die exit speeds of 21–40 m/min, but when AZ80 alloy was extruded under the same conditions, severe hot cracking occurred. This was due to the occurrence of high-melt-point Mg_3_Bi_2_ phases in the BA53 alloy showing higher thermal stability than Mg_17_Al_12_ phases in AZ80 alloy. Because of this feature, it is possible for Bi-modified AZ series alloys to gain outstanding high-temperature mechanical properties. Therefore, Yuan et al. [10] investigated the effects of Bi addition on the microstructure and mechanical properties of as-cast AZ91 alloy, and the result showed that adding 0.5 wt% Bi increased the tensile yield strength and creep resistance significantly at elevated temperatures up to 573 K. In addition, Ren et al. [11] reported that the tensile strength and elongation of as-cast AZ81 alloy reached maximum values when 2 wt% Bi was added at 423 K, compared to other Bi contents. Obviously, the optimum amount of added Bi depends on the type of AZ series alloy. In order to optimize the novel Bi-modified AZ series alloys, Moshver et al. [12] and Li et al. [13] analyzed the microstructure and mechanical properties of as-cast Mg-(3~9 wt%) Al-Zn-Bi alloys over a wide Bi composition range, and found that 1 wt% Bi was suitable content to add for grain refinement and improved strength and ductility. 

Despite the studies mentioned above, so far, no heat-resistant Mg–Al–Zn–Bi alloys in the as-extruded state have been reported. In general, in comparison to the as-cast state, as-extruded alloys exhibit a more homogeneous structure, more refined grain size, stronger texture component, and more plentiful dynamic precipitates. A uniform fine-grained structure is bad for high-temperature strength due to grain boundary sliding (GBS) [14,15]. On the contrary, a certain number of precipitates inhibit the motion of dislocations to some extent, guaranteeing high strength during hot deformation [16,17]. This is expected to reduce GBS and dislocation movement to enhance heat resistance. However, the impact of adding Bi to as-extruded Mg–Al–Zn–Bi alloy during hot deformation is not yet known. 

Therefore, in this work, a new as-cast Mg-3Al-1Zn-3Bi (AZB313, in wt%) alloy was prepared and subsequently treated by solid solution and extruded into bars. AZ31 alloy subjected to the same experimental conditions was used as a benchmark. The impact of Bi addition on the heat resistance of the as-extruded AZ31 alloy was systematically investigated.

## 2. Materials and Methods

The as-cast AZB313 alloy was prepared in an electric resistance furnace (Shanghai Yuzhi Technology Co., Ltd., Shanghai, China) under an atmosphere of mixed SF_6_ and CO_2_ gas (mixing volume ratio was 1:99). The materials used in this study were commercial pure Mg ingot (≥99.99%), pure Al ingot (≥99.9%), pure Zn granules (≥99.9%), and Mg-10Bi (in wt%) and Mg-10 Mn (in wt%) master alloys. The detailed smelting operation can be found our previous work [18]. The chemical composition of the as-cast alloy was detected by X-ray fluorescence spectrometry (XRF; LAB CENTER XRF-1800, Shimadzu, Japan), and the results were as follows: 93.1 wt% Mg, 2.8 wt% Al, 0.9 wt% Zn, 2.9 wt% Bi, 0.3 wt% Mn. Subsequently, the as-cast AZB313 alloy was subjected to solid solution treatment at 773 K for 12 h, then extruded into bars with an extrusion ratio of 30:1 and a die exit speed of 4 mm/s at 623 K. The extrusion process was carried out at XJ-500 horizontal extruder (WuxiYuanchang Machine Manufacture Co., Wuxi, China). Commercial as-cast AZ31 alloy (including 95.9 wt% Mg, 2.9 wt% Al, 0.9 wt% Zn, 0.3 wt% Mn) under the same extrusion conditions was used as a benchmark.

The microstructure of these alloys was observed by scanning electron microscopy (SEM; Gemini SEM 300, Carl Zeiss, Oberkochen, Germany) equipped with energy dispersive spectrometer (EDS; Carl Zeiss, Oberkochen, Germany), X-ray diffraction (XRD; Rigaku D/Max 2500, Rigaku Corporation, Tokyo, Japan) using Cu Kα radiation at a wavelength of 0.15406 nm, and electron backscattered diffraction (EBSD; JEOL JSM-7800F, Japan Electronics Corporation, Tokyo, Japan). EBSD preparation consisted of grinding, washing, blow-drying, and electro-polishing at a voltage of 20 V and an electric current of 0.03 A for 90 s at a temperature of −298 K with a special electrolyte named AC2. The step size of EBSD scanning of all samples was set as 1 μm. All EBSD data were analyzed using ATEX software v2.01.3 (ATEX, Metz, France). Grain size distribution was statistically measured by quantitative metallography method.

A high-temperature annealing process was implemented at 703 K for 0.5, 3, and 5 h using Muffle furnace (Shanghai Yiheng Scientific Instrument Co., Ltd., Shanghai, China). The grain growth rate during high-temperature annealing can be calculated as follows:

(1) $$\delta =\frac{d_{l}-d_{f}}{\Delta t}$$

where $d_{f}$ and $d_{l}$ represent the average grain sizes of alloy before and after annealing, respectively. Δt is interval time during annealing. Room-/high-temperature (298, 373, 423, 473, and 523 K) compressive properties of all samples with a size of Φ8×12 mm (diameter × height) were measured using a CMT6305-300 kN universal testing machine (MTS Systems (China) Co., Ltd., Shenzhen, China) at a strain rate of 1 × 10^−3^ s^−1^. Each test was performed three times to guarantee the accuracy of experiments. To understand the microstructure evolution of alloys during hot compression, quasi in situ SEM observation was performed by interrupting the process at some specific strain levels. Prior to loading, the SEM image was taken in the center of the gage section of the compressive sample. A micro-hardness (Suzhou Tepurui Electromechanical Technology Co., Ltd., Suzhou, China) indentation was used as a marker to track the same area at different strain levels throughout compression.

## 3. Results and Discussion

### 3.1. Microstructure of as-Cast Alloys

**Figure 1:**
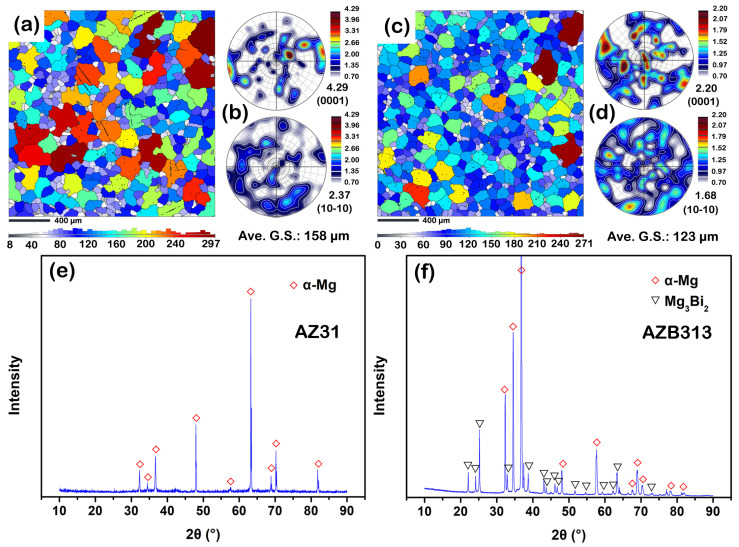
(**a**,**c**) EBSD inverse pole figure maps showing grain size distribution; (**b**,**d**) crystal orientation distribution maps including (0001) and (10-10) pole figures; (**e**,**f**) XRD results of as-cast AZ31 and AZB313 alloys, respectively.

Figure 1 shows the microstructure of the as-cast AZ31 and AZB313 alloys. In Figure 1a,c, each grain can be distinguished clearly by color, where cold-toned colors represent small-sized grains and warm colors indicate large-sized grains. The area fraction of warm-toned grains is larger in the as-cast AZ31 alloy (Figure 1a) than in the as-cast AZB313 (Figure 1c). On the contrary, compared with the as-cast AZ31 alloy, cold-toned grains occupy most of the area in the as-cast AZB313. By statistical analysis, the as-cast AZ31 alloy had a larger average grain size (~158 μm) than the as-cast AZB313 (~123 μm). This result indicates that adding Bi can refine the grains of as-cast AZ series alloys, which is also consistent with the reports of Moshver et al. [12], Li et al. [13], and Wang et al. [19]. During the solidification process, the addition of Bi can be expected to generate a constitutional undercooling diffusion layer ahead of the solid/liquid interface, impeding the diffusion of Mg atoms and thus reducing the growth rate of α-Mg [20]. Figure 1b,d shows the crystal orientation of the two as-cast alloys. There are no obvious textural features from the observation results of (0001) and (10-10) pole figures. XRD tests were performed to measure the phase constitution of the two alloys. For the as-cast AZ31 alloy (Figure 1e), only the diffraction peaks of α-Mg could be detected. However, besides α-Mg, the Bi addition made Mg_3_Bi_2_ phases form in the as-cast AZB313 alloy (Figure 1f).

**Figure 2:**
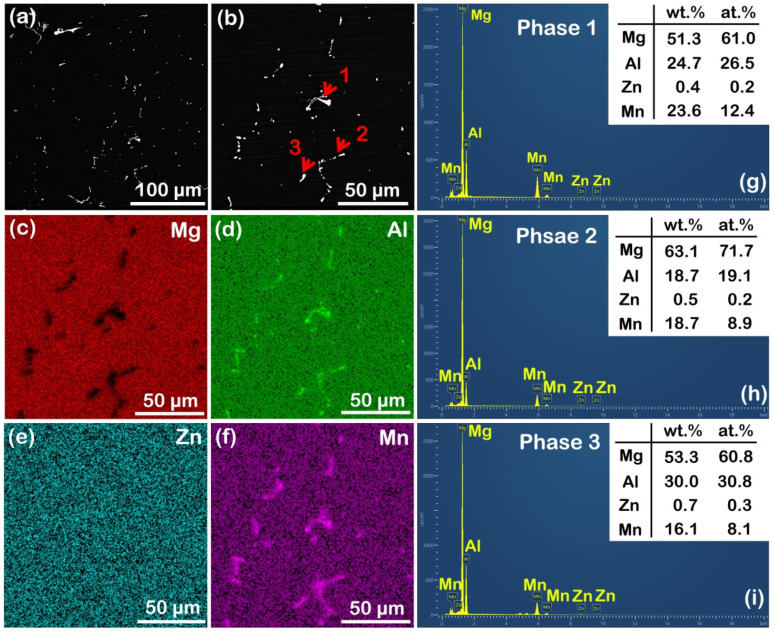
SEM analysis of as-cast AZ31 alloy: (**a**) SEM image; (**b**) magnified SEM image; (**c**–**f**) SEM mapping results showing Mg, Al, Zn, and Mn distributions; (**g**–**i**) SEM point scanning results of phases 1–3 marked by red arrows in (**b**), respectively.

Figure 2 shows the SEM results of the as-cast AZ31 alloy. It still had a small number of irregular bright second phases (Figure 2a,b), mainly composed of Al and Mn according to the SEM mapping (Figure 2c–f). Combining the analysis results of SEM point scanning of phases 1–3 (marked by red arrows in Figure 2b) in Figure 2g–i, we can confirm that these phases were Al_8_Mn_5_. It has been widely reported that the element Mn is often added to Mg alloys to purify the melt; meanwhile, the interaction of Al and Mn forms Al_8_Mn_5_ phases retained in alloys [21]. However, they were barely measured during the XRD test (Figure 1e), probably because of their low area fraction.

**Figure 3:**
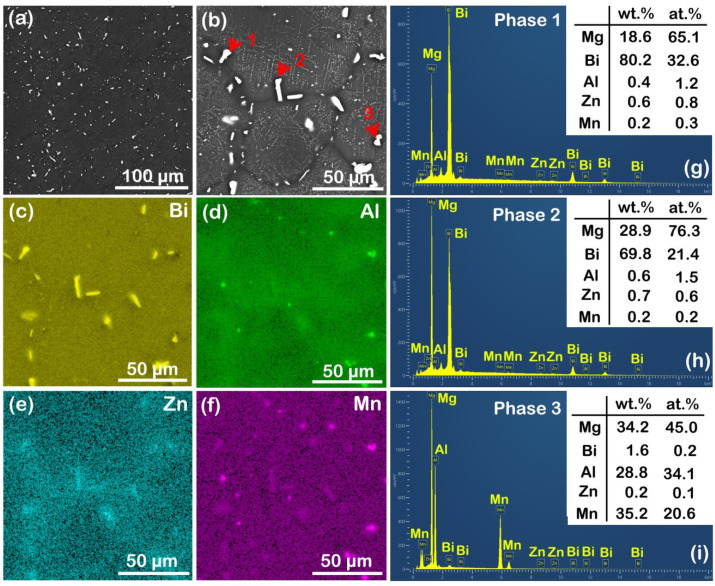
SEM analysis of as-cast AZB313 alloy: (**a**) SEM image; (**b**) magnified SEM image; (**c**–**f**) SEM mapping results showing Bi, Al, Zn, and Mn distributions; (**g**–**i**) SEM point scanning results of phases 1–3 marked by red arrows in (**b**), respectively.

In comparison to the as-cast AZ31 alloy, the as-cast AZB313 alloy presented different second-phase characteristics (Figure 3). Observed at low magnification (Figure 3a), some approximately granular-shaped phases can be seen distributed on the matrix. The view at higher magnification shows that there are two types of phases, large-sized granular-shaped divorced eutectic phases mainly concentrated in the grain boundaries and small-sized needle-shaped phases dispersed within the grains (Figure 3b). This kind of phase distribution was not seen in the as-cast AZ31 alloy. Based on the SEM mapping (Figure 3c–f), these large-sized granular-shaped phases were mainly considered to be Al-Mn and Mg-Bi phases, noting that small amounts of Zn and Mn were also detected to enrich the Mg–Bi phases. Our recent work pointed out that the incorporation of Zn in Mg_3_Bi_2_ phases favors the reduction of formation enthalpy to increase their nucleation and number density [20]. Here, the incorporation of Zn and Mn had a similar effect. Together with the SEM point scanning result of phases 1–3 (labeled by red arrows in Figure 3b) shown in Figure 3g–i, it was found that these phases were mainly Al_8_Mn_5_ and Mg_3_Bi_2_. There was an obviously higher area fraction of Mg_3_Bi_2_ phases than Al_8_Mn_5_, thereby making the diffraction peaks of α-Mg and Mg_3_Bi_2_ appear during the XRD test (Figure 1f). As for the small-sized needle-shaped phases, they were regarded as secondary precipitates during the solidification process. As reported in our recent work [20], these secondary precipitates were mainly composed of Mg_3_Bi_2_ phases. In their work, Yu et al. [22] also observed this phenomenon: with increased Bi content, the fine Mg_3_Bi_2_ precipitates gradually increased in Mg–Bi alloys. In addition, we also found that the morphology of Al_8_Mn_5_ phases changed from irregular to granular shape, accompanied by a decrease in phase size. This indicates that the addition of Bi is beneficial for the spherification and refinement of Al_8_Mn_5_ phases. The detailed mechanism is unknown and needs to be studied in future work.

### 3.2. Microstructure of as-Extruded Alloys

Before extrusion, as-cast alloys need to undergo solid solution treatment. The EBSD and SEM results of solid solution treated alloys are shown in Figure S1 (see Supplementary Materials). From the observation of EBSD, the average grain size of the two solid solution treated alloys had an increasing trend, to ~220 and ~142 μm (Figure S1a,b). This indicates that the as-cast AZ31 alloy showed larger grain growth during solid solution treatment than the as-cast AZB313 alloy. According to the SEM results, those irregular Al_8_Mn_5_ phases were retained in the solid solution treated AZ31 alloy, and at the same time, it was hard for the large-sized granular-shaped Mg_3_Bi_2_ and Al_8_Mn_5_ phases to dissolve in the matrix of the solid solution treated AZB313 alloy due to high melting points above 773 K (Figure S1c,d). However, the fine needle-shaped Mg_3_Bi_2_ precipitates were dissolved in the matrix (Figure S1d). Combining the above results, it is expected that the reserved Mg_3_Bi_2_ phases distributed at the grain boundaries provide a barrier against grain boundary migration during solid solution treatment, lowering the grain growth rate of solid solution treated AZB313 alloy.

**Figure 4:**
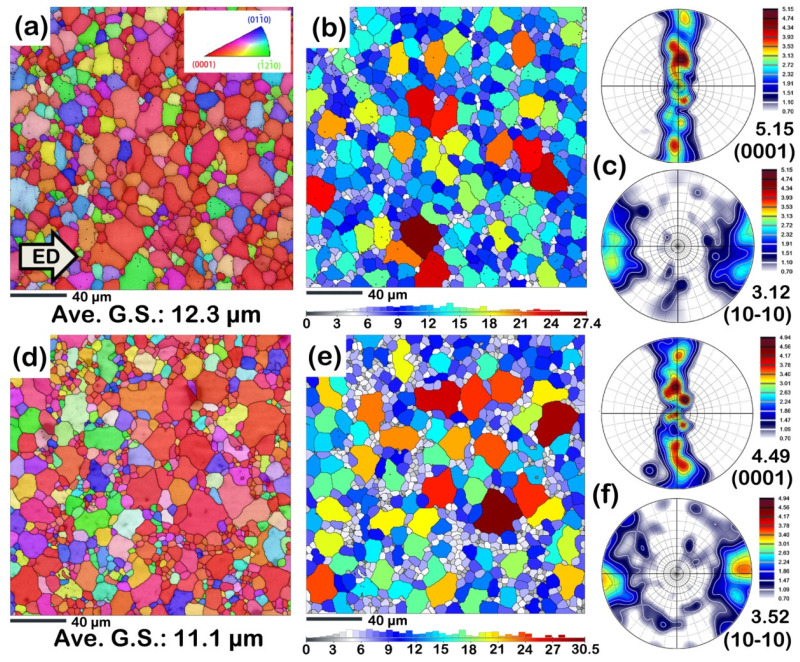
(**a**,**d**) EBSD inverse pole figure maps; (**b**,**e**) EBSD inverse pole figure maps showing grain size distribution; (**c**,**f**) crystal orientation distribution maps including (0001) and (10-10) pole figures for as-extruded AZ31 and AZB313 alloys, respectively.

Figure 4 shows the microstructure of the as-extruded AZ31 and AZB313 alloys. Both alloys exhibited a complete but heterogeneous dynamic recrystallization (DRX) structure (Figure 4a,d), which is typical of as-extruded Mg alloys [23]. From the results of grain size distribution maps (Figure 4b,e), the two as-extruded alloys had similar average grain size (~12.3 and ~11.1 μm). This result indicates that Bi addition has little influence on the grain size of as-extruded AZ31 alloy. With respect to crystal orientation, both as-extruded alloys showed a similar basal fiber texture (Figure 4c,f). The c-axes of most grains were perpendicular to the extrusion direction (ED) in (0001) pole figures, and their <10-10> were parallel to the ED in (10-10) pole figures. The maximum pole intensities were also almost the same. Obviously, the texture formation is independent of the Bi addition.

**Figure 5:**
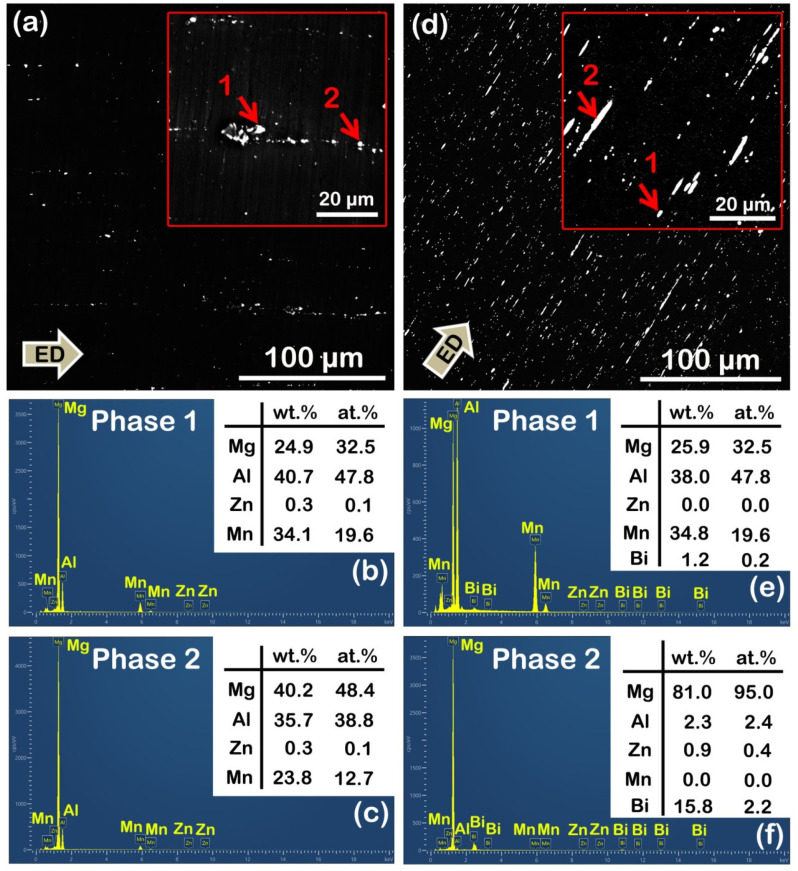
(**a**,**d**) Low-magnification SEM images of as-extruded AZ31 and AZB313 alloys, respectively; (**b**,**c**) SEM point scanning results of phases 1 and 2 marked by red arrows in (**a**); (**e**,**f**) SEM point scanning results of phases 1 and 2 labeled by red arrows in (**b**).

**Figure 6:**
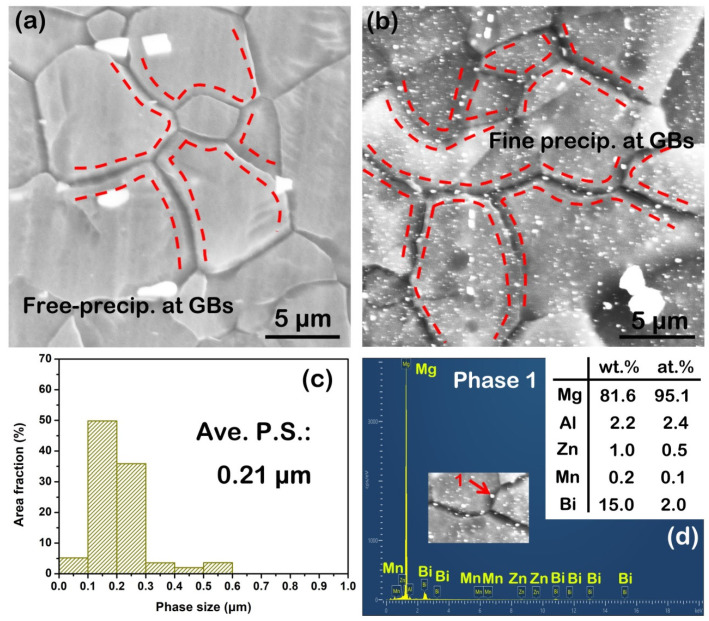
(**a**,**b**) High-magnification SEM images of as-extruded AZ31 and AZB313 alloys, respectively; (**c**) phase size distribution map of fine dynamic precipitates from (**b**); (**d**) SEM point scanning result of phases 1 marked by red arrow.

However, in terms of second phase, low-magnification SEM observation shows that the as-extruded AZB313 alloy had a larger area fraction (~3%) than the as-extruded AZ31 (~0.4%) (Figure 5a,d). These small-sized phases distributed along the ED, with an average size of ~2 μm, were considered to be the products of the breakup of retained large-sized phases after solid solution treatment. Based on the SEM point scanning results, we can further confirm that the phase constitution of the as-extruded AZ31 and AZB313 alloys was still mainly Al_8_Mn_5_ (Figure 5b,c) and Mg_3_Bi_2_ plus Al_8_Mn_5_ (Figure 5e,f). Interestingly, under high magnification, profuse submicron-sized second phases were found to disperse within grains and locate at grain boundaries in the as-extruded AZB313 alloy, but this phenomenon was not found in the as-extruded AZ31 (Figure 6a,b). These phases can be attributed to dynamic precipitation. By statistical analysis, the average size of these fine dynamic precipitates was ~0.21 μm (Figure 6c). According to the SEM point scanning result of phase 1, it was verified as Mg_3_Bi_2_ phase (Figure 6d). According to the equilibrium phase diagram for Mg-*x*Al-3Bi (*x* = 0–4 wt%) alloy [8], at an extrusion temperature of 350 °C, dynamic precipitation of fine Mg_3_Bi_2_ phases could occur during extrusion. In addition, Mg_17_Al_12_ phase can theoretically be formed below an extrusion temperature of 200 °C in as-extruded Mg-3Al-3Bi alloy, but after the material exits the extrusion die, it is quickly air-cooled to room temperature, and consequently there is not enough time for Al solute atoms to diffuse for the formation of Mg_17_Al_12_ phases [8]. This is a fairly good confirmation of our result.

It is well known that dispersed fine precipitates can hinder the growth of grains during extrusion, based on the theory of Zener drag [24,25]. However, in this work, even though we observed abundant submicron-sized Mg_3_Bi_2_ dynamic precipitates in the as-extruded AZB313 alloy (Figure 6b), the average grain size was still similar to that of the as-extruded AZ31 alloy (Figure 4b,e). Such a difference may be related to the order of dynamic precipitation. If dynamic precipitation occurs prior to DRX during extrusion, fine precipitates will largely inhibit the formation of DRXed grains and grain boundary motion. This agrees with the theory of Zener drag. On the contrary, if DRX occurs preferentially during extrusion, the DRXed grains grow quickly under the extrusion heat. Then, Mg_3_Bi_2_ phases are dynamically precipitated within the grown grains and their boundaries. In this case, the effect of fine dynamic precipitates on further restricting the growth of DRXed grains is limited. Therefore, we can conclude that DRX may be preferred over dynamic precipitation of fine Mg_3_Bi_2_ phases during extrusion with Bi addition, weakening the Zener drag effect and resulting in a similar average grain size in both as-extruded alloys. To a certain extent, the degree of DRX determines the texture during hot deformation [26,27]. Hence, under the conditions of similar DRX behavior, both as-extruded alloys present almost the same basal fiber texture (Figure 4c,f).

### 3.3. Heat Resistance of as-Extruded Alloys

#### 3.3.1. “Static” Heat Resistance

**Figure 7:**
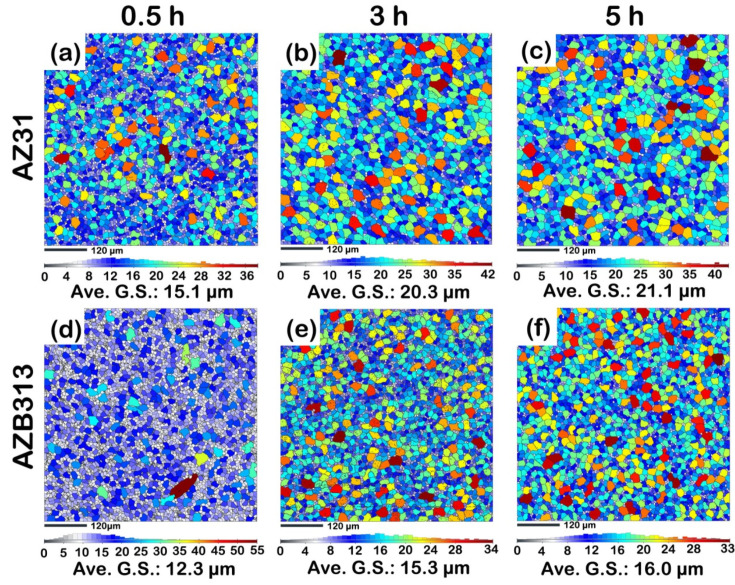
EBSD inverse pole figure maps showing grain size distribution during high-temperature annealing at 773 K for (**a**,**d**) 0.5 h; (**b**,**e**) 3 h; and (**c**,**f**) 5 h in as-extruded AZ31 and AZB313 alloys, respectively.

**Figure 8:**
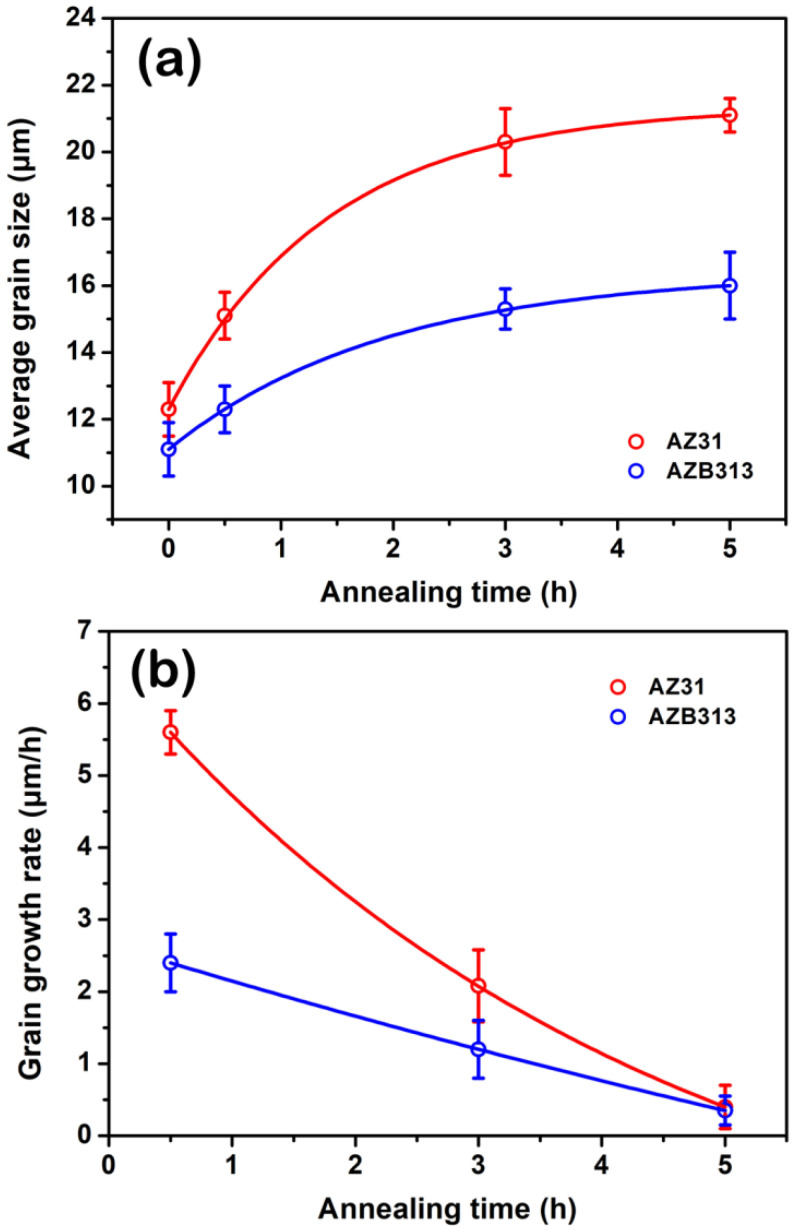
(**a**) Average grain size and (**b**) grain growth rate as a function of annealing time during high-temperature annealing in two as-extruded alloys.

Figure 7 shows the evolution of average grain size of as-extruded AZ31 and AZB313 alloys during high-temperature annealing at 703 K for 0.5, 3, and 5 h. It can be seen that with extended annealing time, the average grain size gradually increased in both alloys. With annealing for 0.5 h, the average grain size increased to ~15.1 and ~12.3 μm, respectively (Figure 7a,d). The corresponding grain growth rates were ~5.60 and ~2.40 μm/h, respectively. With annealing time of 3 h, the average grain size continuously grew to ~20.3 and ~15.3 μm, respectively (Figure 7b,e). At this point, the corresponding grain growth rates were as slow as ~2.08 and ~1.20 μm/h, respectively. Until 5 h, the rising trend for grain size was increasingly inconspicuous. The average grain size was only ~21.1 and ~16.0 μm (Figure 7c,f), and the corresponding growth rates decreased to ~0.40 and ~0.35, respectively. The measurements were plotted as curves, shown in Figure 8. Compared with the as-extruded AZ31 alloy, the as-extruded AZB313 alloy shows lower average grain size and grain growth rate during high-temperature annealing, indicating that the Bi addition can remarkably provide high “static” heat resistance to impede the growth of grains.

As is known, the growth of grains is closely associated with grain boundary curvature [28], storage energy [29,30], misorientation [31], and second phase [24,25,32]. In general, curved grain boundaries tend to flatten. The boundaries of small-sized grains have a larger curvature to provide a driving force for grain boundary migration [28]. However, in this work, grain boundary curvature was not a crucial factor influencing the difference in grain growth rates for the two as-extruded alloys, since they had similar average grain size. In addition, Wu et al. [30] found that grains with [2-1-11]//ED grew preferentially because of a lower kernel average misorientation (KAM) value than grains with [10-10]//ED in Mg-1Gd (in wt%) alloy, and they believed that the difference in storage energy was the reason for this result. Clearly, this theory was not applied to our work, because both as-extruded alloys showed a complete DRX structure with extremely low storage energy (Figure S2, see Supplementary Materials). Similarly, some studies have reported that differences in misorientation can also affect grain growth because of the anisotropy in grain boundary properties, including energy and mobility [31]. Figure S3 (see Supplementary Materials) shows the orientation maps of both as-extruded alloys, where it can be seen that there is no distinction at all. Therefore, it is reasonable to believe that misorientation also had little impact on the difference in grain growth rates for the two as-extruded alloys. 

According to the principle of Zener drag [24,25], the migration of grain boundaries will be hindered if the resistance force caused by second phases is higher than the driving force of grain growth. In this case, there must be a critical gain size ($D_{c}$). If the average grain size (*D*) of the alloy is larger than $D_{c}$, Zener drag will occur. In contrast, when *D* is smaller than $D_{c}$, Zener drag will not occur. $D_{c}$ can be given as follows [33]:

(2) $$D_{c}=4r/3f$$

where *r* and *f* are the average radius and area fraction of second phases, respectively. In the as-extruded AZ31 alloy, the *r* and *f* of Al_8_Mn_5_ phases were ~1 μm and ~0.4% (Figure 5a), and thus the calculated $D_{c}$ value was ~333.3 μm, far beyond the value of *D* (~12.3 μm). Thus, no Zener drag effect occurred. For the as-extruded AZB313 alloy, we had to separately calculate the roles of the two types of second phases on the effect of Zener drag. For large-sized phases, including Mg_3_Bi_2_ and Al_8_Mn_5_, derived from the breakup of non-dissolved phases, *r* and *f* were ~1 μm and ~3%, respectively (Figure 5b), and $D_{c}$ was determined as ~44.4 μm; this value still exceeds the value of *D* (~11.1 μm) which means these large-sized phases had little influence on the effect of Zener drag. However, for submicron-sized Mg_3_Bi_2_ dynamic precipitates, *r* and *f* of these phases were ~0.1 μm and ~2%, respectively (Figure 6b,c), and $D_{c}$ was calculated as ~6.7 μm, which is smaller than the value of *D* (~11.1 μm). Obviously, Zener drag will occur, impeding the migration of grain boundaries. Therefore, in this work, profuse fine precipitates played an important role in inhibiting grain growth and reducing the grain growth rate of the as-extruded AZB313 alloy to obtain higher “static” heat resistance than the as-extruded AZ31 alloy during high-temperature annealing.

**Figure 9:**
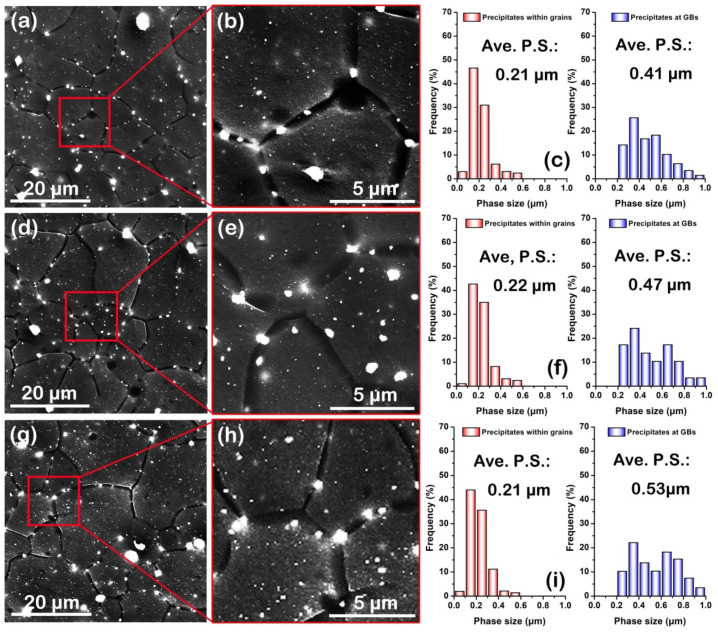
SEM images and phase size distribution maps of precipitates (including within grains and at grain boundaries) in as-extruded AZB313 alloy after high-temperature annealing for (**a**–**c**) 0.5, (**d**–**f**) 3, and (**g**–**i**) 5 h.

It is important to emphasize that whether these precipitates can maintain the original fine size at high temperature is the key to ensuring high “static” heat resistance. Therefore, we observed the evolution of average phase size of precipitates in the as-extruded AZB313 alloy during high-temperature annealing, as shown in Figure 9. After 0.5 h, there were still massive precipitates distributed within grains and at grain boundaries (Figure 9a). Under high magnification (Figure 9b), the precipitates within grains were found to maintain the original size of ~0.21 μm, but the average phase size of those dispersed at grain boundaries slightly grew to ~0.41 μm based on the statistical analysis (Figure 9c). This phenomenon is the so-called grain boundary partitioning effect of solutes [34]. Grain boundaries can act as channels for the diffusion of elements, thereby making original fine precipitates assemble and produce clusters at high temperature. With extended annealing time of 3 and 5 h, these precipitates never dissolved in the matrix (Figure 9d,g). No signs of growth of precipitates within grains were found, since the average phase size was still ~0.21 μm after 3 h (Figure 9e,f) and 5 h (Figure 9h,i). Although there was an uptrend in average phase size of those at grain boundaries, the increment was limited (Figure 9e,h). Until 5 h, it increased to only ~0.53 μm (Figure 9i). As mentioned above, with the addition of Bi, the newly developed Mg_3_Bi_2_ dynamic precipitates presented relatively better thermostability in the as-extruded AZB313 alloy, thereby showing higher “static” heat resistance than the as-extruded AZ31 alloy during high-temperature annealing.

#### 3.3.2. “Dynamic” Heat Resistance

**Figure 10:**
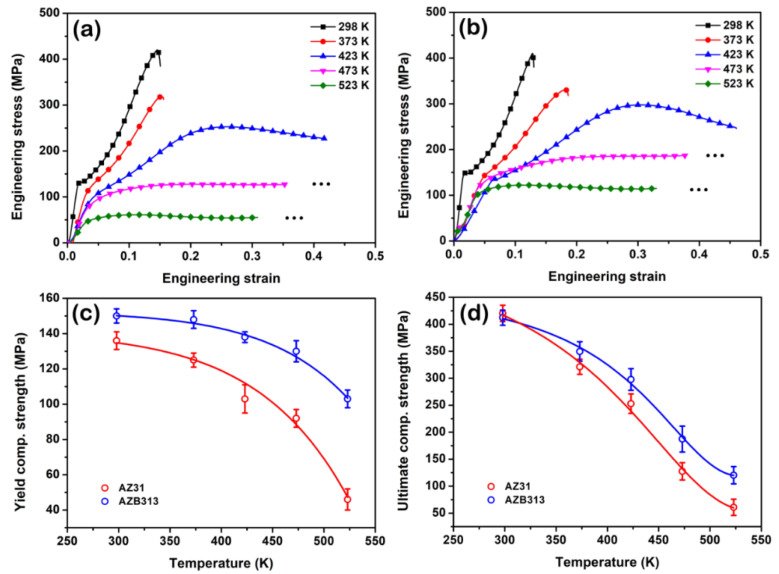
(**a**,**b**) Compressive engineering stress–strain curves of as-extruded AZ31 and AZB313 alloys, respectively, along ED at different temperatures; (**c**,**d**) comparison between compressive yield (and ultimate) strength values of as-extruded alloys, respectively.

In addition to “static” heat resistance, in this section we focus on the analysis of the influence of Bi addition on the “dynamic” heat resistance of the as-extruded AZ31 alloy. Figure 10a,b shows the compressive engineering stress–strain curves of as-extruded AZ31 and AZB313 alloys along the ED at different temperatures. The corresponding compressive yield and ultimate strength values are plotted in Figure 10c,d. It is obvious that the S-shaped curve gradually transitions to a convex curve for both as-extruded alloys with increased compressive temperature. Such an S-shaped curve has been widely reported as a typical indicator of twinning deformation at room temperature [35]. In this work, the basal texture features of both as-extruded alloys showed a “soft” orientation for extension twinning {10–12}. As the compressive temperature rises, the critical resolved shear stress of slip systems decreases remarkably, causing the activation of massive slip dislocations [16,36]. Above 473 K, slip deformation dominates the compressive process (convex curve). Looking at the yield and ultimate strength values in Figure 10c,d, in general, both strength values tended to decline with deformation temperature. When compressed at room temperature (298 K), the as-extruded AZB313 alloy had a slightly higher yield strength (~150 MPa) than the as-extruded AZ31 (~138 MPa), but they had a similar ultimate strength value (~410 MPa). With increasing compressive temperature, the decrement between strength values of the two as-extruded alloys was increasingly large; the maximum differences were ~60 and ~70 MPa for yield and ultimate strength, respectively, at 523 K.

As is known, grain size, texture, and second phase determine the mechanical properties of Mg alloys at room temperature to some extent. In this work, the Bi addition had little impact on the grain size and texture of the as-extruded AZ31 alloy (Figure 4), but a certain amount of fine Mg_3_Bi_2_ phase was dynamically precipitated (Figure 6). This result indicates that only second phase strengthening provided an increment in strength induced by the Bi addition in the as-extruded AZB313 alloy at 298 K, rather than grain boundary and texture strengthening. According to the law of Orowan strengthening [37], the theoretical increment in yield strength from Mg_3_Bi_2_ dynamic precipitates can be estimated at ~17 MPa, which is similar to the experimental value (~12 MPa). 

With increasing deformation temperature, profuse slip dislocations are activated, which decreases the texture dependence of high-temperature strength [38]. Xie et al. [39] reported that $\sigma_{0}$ and $k_{y}$ (which represent the friction stress and Hall–Petch slope, respectively, in the Hall–Petch relationship) were functions of the deformation temperature for as-extruded Mg-0.8 Ca-0.5 Mn (in wt%) based alloy, and with increased T and $T_{m}$ (deformation temperature and melting point, respectively), both constants showed a rapid linear descent. This suggests that the yield strength contribution of grain boundary strengthening is dramatically reduced during deformation at high temperature. In terms of the effect of second phase, Ganeshan et al. [40] reported that there was only a slight decrease in the elastic properties of Mg-rich phases with rising temperature from 298 to 523 K. Hence, the yield strength increment via second phase strengthening at high temperature is considered to be the same as that at room temperature. 

As discussed above, we can speculate that the difference in yield strength between the two as-extruded alloys should theoretically be ~12 MPa at 523 K, which is mainly derived from the contribution of second phase strengthening. However, the experimental value (~60 MPa, at 523 K) greatly exceeds this theoretical value. Obviously, there are extra factors enhancing the “dynamic” heat resistance of the as-extruded AZ31 alloy by the addition of Bi. 

**Figure 11:**
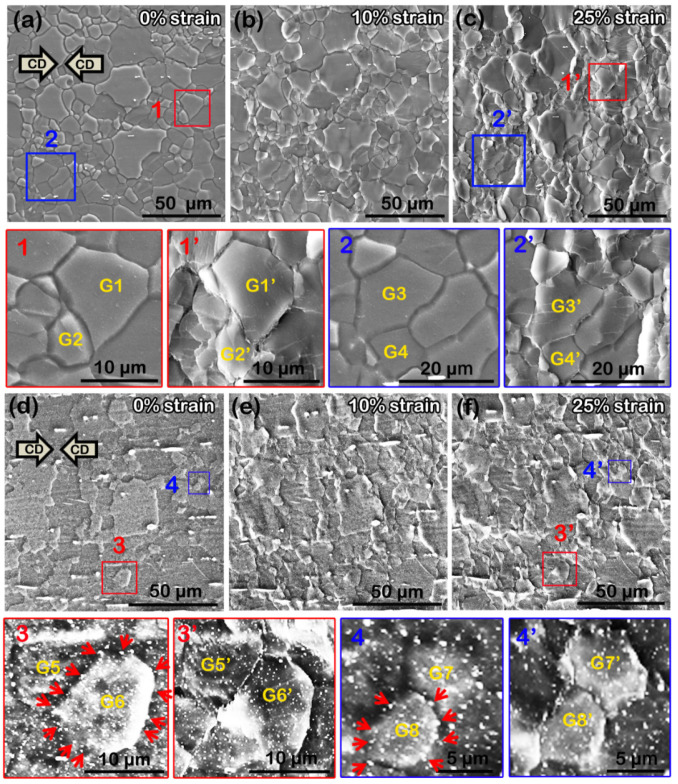
Quasi in situ SEM images of two as-extruded alloys subjected to hot compression at different strains: 0, 10, and 25% strains for (**a**–**c**) as-extruded AZ31 alloy and (**d**–**f**) as-extruded AZB313 alloy, respectively.

**Figure 12:**
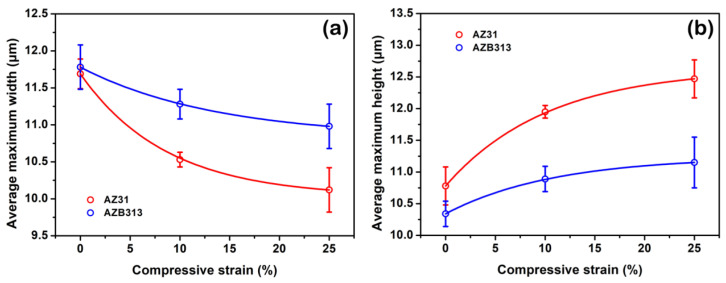
(**a**) Average maximum width of grains (along CD) as a function of compressive strain; (**b**) average maximum height of grains (perpendicular to CD) for as-extruded AZ31 and AZB313 alloys.

To analyze this phenomenon, the microstructural evolution of both as-extruded alloys during compression at 523 K was observed by quasi in situ SEM (Figure 11). Figure 11a–c shows the quasi in situ SEM images of the as-extruded AZ31 alloy subjected to compression at 0, 10, and 25% strains, respectively. It can be seen that with increasing compressive strain, the morphology of many grains changed: they shrunk along the CD and elongated perpendicular to the CD. For example, measurement shows that the maximum width (along the CD) of G1 (and G2) grain decreased from ~15.56 to ~13.88 μm (~7.32 to ~6.53 μm) and the maximum height (perpendicular to the CD) increased from ~17.38 to ~18.08 μm (~9.40 to ~11.69 μm) when the compressive strain increased from 0 to 25%. A similar tendency was also observed in G3 and G4 grains, and the corresponding data are listed in Table 1. However, for the as-extruded AZB313 alloy, with increased compressive strain from 0 to 25%, the vast majority of grains maintained the original morphology (Figure 11d–f). For instance, we observed that the maximum width (along the CD) of G5 (and G6) grain slightly reduced from ~10.33 to ~10.01 μm (~11.68 to ~11.45 μm), and the maximum height (perpendicular to the CD) between G5 (and G6) and G5′ (and G6′) grains was almost the same (~9.62 and ~11.87 μm, respectively). The observations for G7 and G8 grains were similar, as seen in Table 1. Through statistical analysis (100 grains selected randomly), the average maximum width and height of both as-extruded alloys at compressive strains of 0, 10, and 25% were determined, which are summarized in Table 2 and plotted in Figure 12. It can be clearly seen that the variation degree of average maximum width and height of grains was smaller for the as-extruded AZB313 alloy than the as-extruded AZ31 during hot compression. This indicates that the addition of Bi effectively hindered the deformation of grains, further enhancing the thermal stability of the grain structure. By careful observation, we found that fine dynamic precipitates were dispersed around the grain boundaries of these grains (G5–G8). It has been reported that the segregation of second phases (or solutes) at grain boundaries can remarkably reduce grain boundary energy [18,33,39,41]. Here, such a thermally stable grain structure was mainly due to the formation of fine Mg_3_Bi_2_ phases distributed at grain boundaries, hindering the grain rotation.

**Figure 13:**
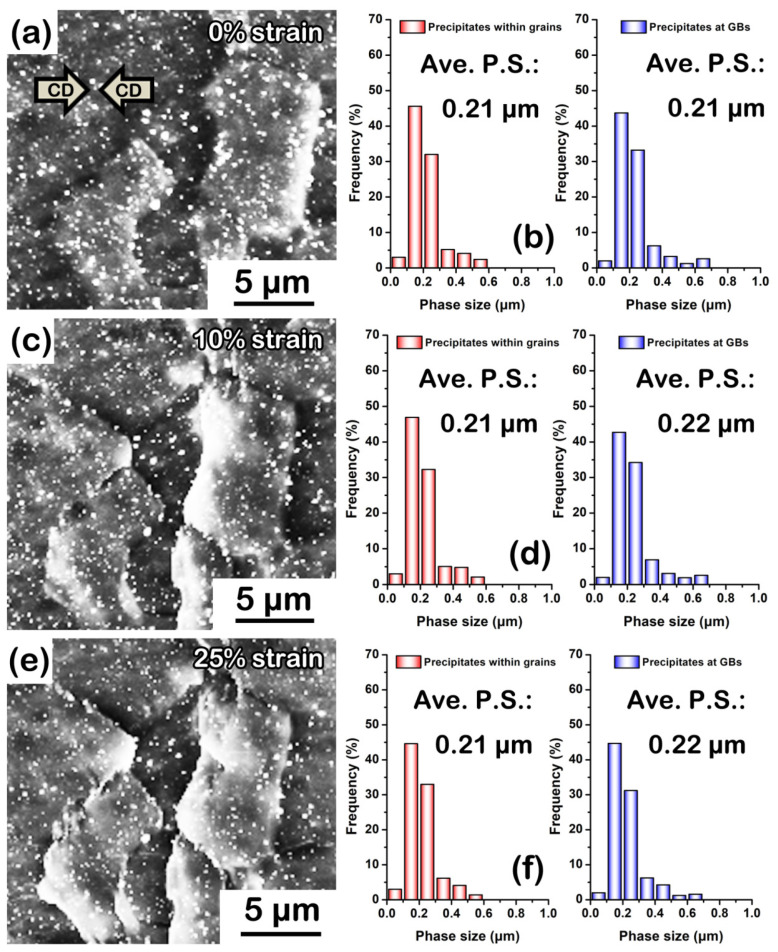
Quasi in situ SEM images and phase size distribution maps of precipitates (including within grains and at grain boundaries) in as-extruded AZB313 alloy after hot compression at 523 K at (**a**,**b**) 0%, (**c**,**d**) 10%, and (**e**,**f**) 25%.

Of course, we also had to consider the thermal stability of the fine Mg_3_Bi_2_ phases during hot compression. The evolution of average phase size of precipitates in the as-extruded AZB313 alloy during hot compression at 523 K is shown in Figure 13. By quasi in situ SEM observation, no obvious change was found with strains from 0 to 25% (Figure 13a,c,e). The measured results also confirm that Mg_3_Bi_2_ phases were thermally stable, since their average phase size (within grains and at grain boundaries) stayed almost the same at different strains (Figure 13b,d,f).

**Figure 14:**
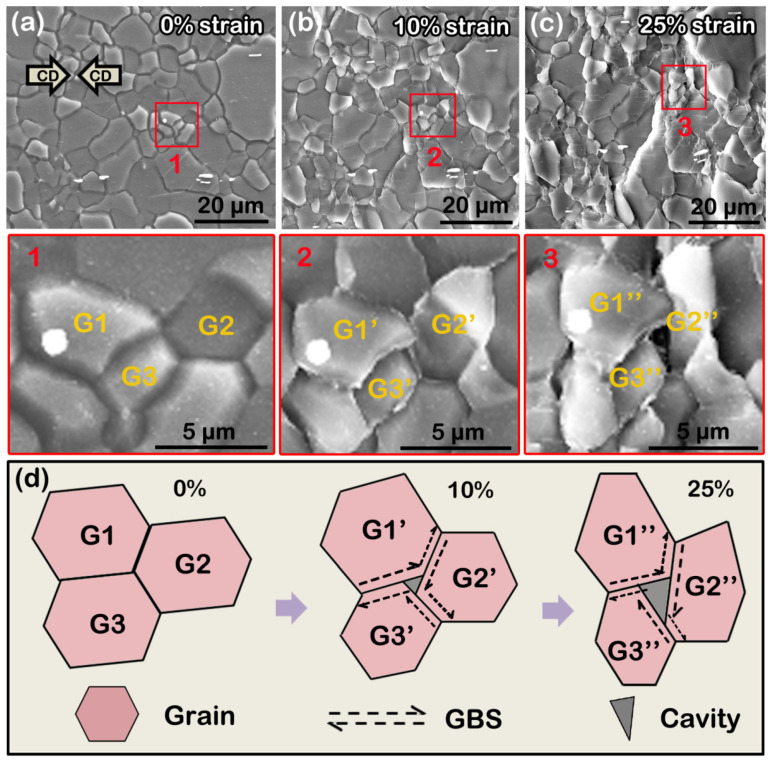
Quasi in situ SEM images of as-extruded AZ31 alloy subjected to hot compression at different strains: (**a**–**c**) 0, 10, and 25% strain, respectively. (**d**) Schematic diagram of formation and propagation of GBS-induced cavity.

GBS is generally deemed to be an important mechanism to obtain high ductility, even super-plasticity, further deteriorating the strength [14,15]. Fine grain size, high deformation temperature, and low strain rate are beneficial for its occurrence. In general, the forming temperature of GBS is above 0.5 $T_{m}$. However, recent studies have reported that it can be activated in ultra-fine-grained Mg alloys during tension (or compression) with an extremely low strain rate at room temperature [42,43]. If the grains are microcrystalline, the temperature required for GBS to occur is only ~0.32 $T_{m}$ (~480 K for Mg alloys) [44]. A typical feature of GBS is a tendency for obvious cavities at the trigeminal boundaries. In this work, we observed signs of GBS at local fine-grained areas in the as-extruded AZ31 alloy after hot compression at 523 K (Figure 14). We selected a typical region and subsequently revealed the GBS process within this area during hot compression by quasi in situ SEM observation. The high-magnification views of regions 1–3 (corresponding to 0, 10, and 25% strains in Figure 14a–c, respectively) show the formation and propagation of a GBS-induced cavity. This process is described in Figure 14d. With increased strain from 0 to 10%, the trigeminal boundaries of G1–G3 grains slid against each other and initially formed a small, triangle-like cavity. When the strain increased to 25%, the cavity enlarged and propagated at the grain boundaries, since the degree of GBS gradually strengthened. Thus, for the as-extruded AZ31 alloy, the occurrence of GBS mainly limited to fine-grained areas was another factor in reducing the high-temperature strength compared with the as-extruded AZB313 alloy, in which this was effectively restricted.

#### 3.3.3. Heat Resistance Mechanisms

**Figure 15:**
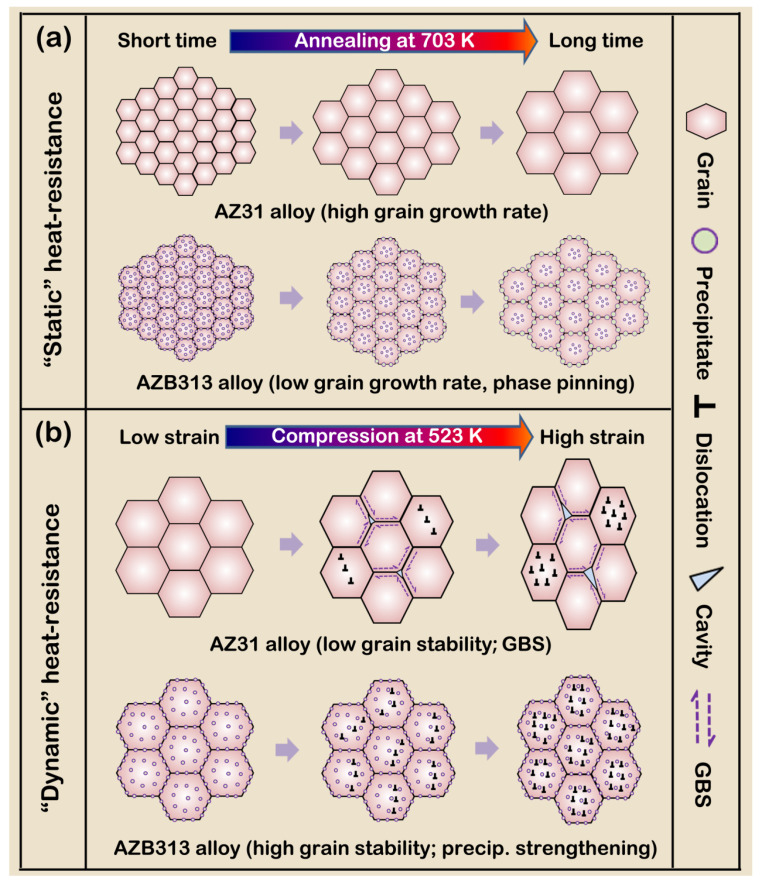
(**a**) “Static” and (**b**) “dynamic” heat resistance mechanisms of both as-extruded AZ31 and AZB313 alloys.

As mentioned above, we have systematically analyzed the thermal-stability at high-temperature annealing and the mechanical properties (especially for the strength) at high temperature of as-extruded AZ31 and AZB313 alloys, and conclude that the Bi addition can enhance the “static” and “dynamic” heat resistance of the as-extruded AZ31 alloy. In this section, we draw a schematic diagram (Figure 15) of the “static” and “dynamic” heat resistance mechanisms of the investigated alloy by the Bi addition for readers to better understand.

Figure 15a shows the difference of “static” heat resistance between both the as-extruded alloys with similar grain sizes and texture features. With the Bi addition, fine Mg_3_Bi_2_ phases act as barriers to restrict the grain boundary migration and reduce the grain growth rate during high-temperature annealing for short time. When the annealing time prolongs, these precipitates dispersed at grain boundaries slightly enlarge and weaken the pinning effect of precipitates to a certain extent. Even so, the as-extruded AZB313 alloy still has the higher “static” heat resistance, compared with the as-extruded AZ31 alloy.

Under thermodynamic coupling conditions, as shown in Figure 15b, the as-extruded AZ31 alloy exhibits the obvious deformation of grain via dislocation slip and local GBS, lowering the grain stability. Nevertheless, for the as-extruded AZB313 alloy, the average phase size of fine Mg_3_Bi_2_ phases never changes during hot compression with the increase of strain. These fine precipitates within grains can effectively hinder the movement of dislocations, thereby increasing the resistance to deformation of grains. Except for precipitation strengthening, high grain stability is also a crucial factor to maintain the strength of the as-extruded AZB313 alloy at high temperature. Furthermore, limited GBS is found, since the pinning effect of the fine precipitates on grain boundaries is very strong, further restricting the occurrence of GBS. Hence, the as-extruded AZB313 alloy presents a higher “dynamic” heat resistance than the as-extruded AZ31 one.

## 4. Conclusions

In this work, the influence of Bi addition on the heat resistance of the as-extruded AZ31 alloy during high-temperature annealing and hot compression was systematically revealed. The main conclusions were listed as follows:

(1) During high-temperature annealing at 703 K, the as-extruded AZB313 alloy had a lower grain growth rate than the as-extruded AZ31, since the Mg_3_Bi_2_ phases (distributed at grain boundaries) impeded the grain boundary motion (even though these precipitates were slightly coarsened) providing higher “static” heat resistance.
(2) During hot compression at 298 to 523 K, the yield and ultimate strength of both as-extruded AZ31 and AZB313 alloys gradually decreased, but with the addition of Bi, this tendency could be delayed. The main reasons include the following aspects: (i) strengthened precipitation—the strength contribution of fine Mg_3_Bi_2_ phases at room temperature could be maintained at high temperature; (ii) the high thermal stability of the grains—the thermally stable Mg_3_Bi_2_ precipitates (dispersed within grains) effectively hindered the movement of dislocations, thereby increasing the resistance to grain deformation; (iii) restricted occurrence of GBS—the pinning effect of the fine precipitates on grain boundaries was very strong, further limiting GBS. Therefore, it was expected that the Bi addition also promoted the enhancement of “dynamic” heat resistance of the as-extruded AZ31 alloy.

## Figures and Tables

**Table 1:** Maximum width (along CD) and height (perpendicular to CD) of G1-G8 grains marked in Figure 11 after hot compressive strain of 0 and 25% at 523 K. Unit: μm.

Grain	Strain	Maximum Width	Maximum Height
G1	0%	15.56 ± 0.22	17.38 ± 0.34
	25%	13.88 ± 0.32	18.08 ± 0.12
G2	0%	7.32 ± 0.24	9.40 ± 0.15
	25%	6.53 ± 0.21	11.69 ± 0.28
G3	0%	19.12 ± 0.15	18.75 ± 0.32
	25%	16.85 ± 0.18	21.01 ± 0.23
G4	0%	8.93 ± 0.23	8.62 ± 0.09
	25%	7.04 ± 0.27	9.98 ± 0.21
G5	0%	10.33 ± 0.32	9.62 ± 0.24
	25%	10.01 ± 0.25	9.68 ± 0.11
G6	0%	11.68 ± 0.14	11.87 ± 0.25
	25%	11.45 ± 0.31	11.92 ± 0.18
G7	0%	5.84 ± 0.11	7.25 ± 0.24
	25%	5.82 ± 0.13	7.30 ± 0.12
G8	0%	6.13 ± 0.32	6.44 ± 0.15
	25%	5.87 ± 0.14	6.50 ± 0.22

**Table 2:** Average maximum width (along CD) and height (perpendicular to CD) of 100 randomly selected grains from as-extruded AZ31 and AZB313 alloys after hot compressive strain of 0, 10, and 25% at 523 K. Unit: μm.

Alloy	Strain	Ave. Maximum Width	Ave. Maximum Height
As-extruded AZ31	0%	11.69 ± 0.21	10.78 ± 0.33
	10%	10.53 ± 0.12	11.95 ± 0.14
	25%	10.12 ± 0.31	12.47 ± 0.32
As-extruded AZB313	0%	11.78 ± 0.32	10.34 ± 0.25
	10%	11.28 ± 0.26	10.89 ± 0.23
	25%	10.98 ± 0.34	11.15 ± 0.42

## Data Availability

Data available on request.

## Supplementary Materials

The following supporting information can be downloaded at: https://www.mdpi.com/article/10.3390/ma16030996/s1. Figure S1. (a,b) EBSD inverse pole figure maps showing grain size distribution; (c,d) SEM results of the solid-solution treated AZ31 and AZB313 alloys, respectively. Figure S2. (a,b) KAM maps; (c,d) KAM histogram maps of the as-extruded AZ31 and AZB313 alloys, respectively. Figure S3. (a,b) Misorientation angle distribution maps of the as-extruded AZ31 and AZB313 alloys, respectively.
