# Supplementary material for: Effect of Bi Addition on the Heat Resistance of As-Extruded AZ31 Magnesium Alloy
Source: Materials (Basel). 2023 Jan 21;16(3):996. doi: 10.3390/ma16030996 (PMC9919633; doi:10.3390/ma16030996)
Supplement: Supplementary file 1 [file materials-16-00996-s001.zip › materials-2155588-supplementary.pdf]

materials

MDPI

Supplementary Materials

Effect of Bi Addition on the Heat Resistance of as-Extruded AZ31 Magnesium Alloy

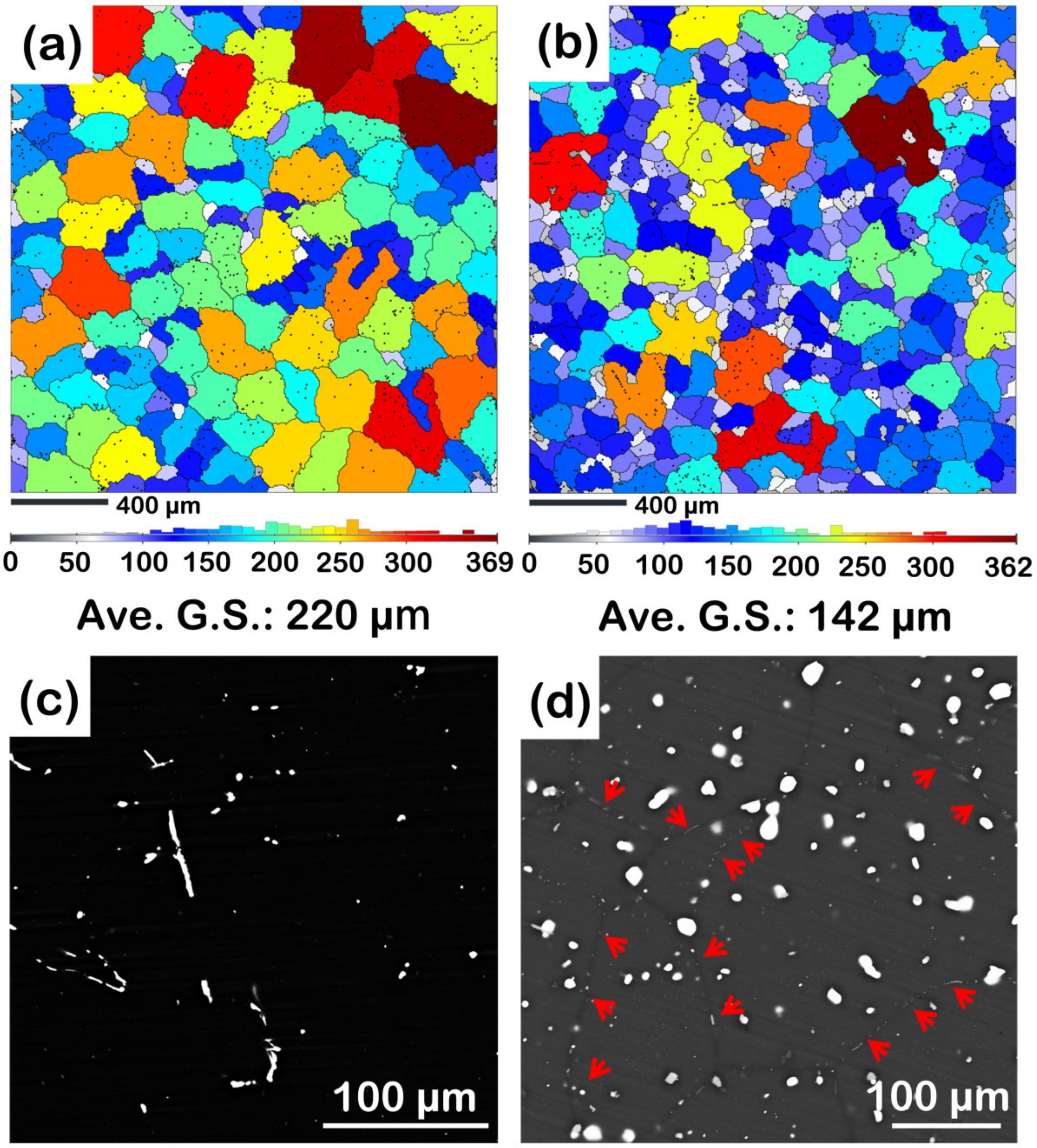

Figure S1. (**a**,**b**) EBSD inverse pole figure maps showing grain size distribution; (**c**,**d**) SEM results of the solid-solution treated AZ31 and AZB313 alloys, respectively.

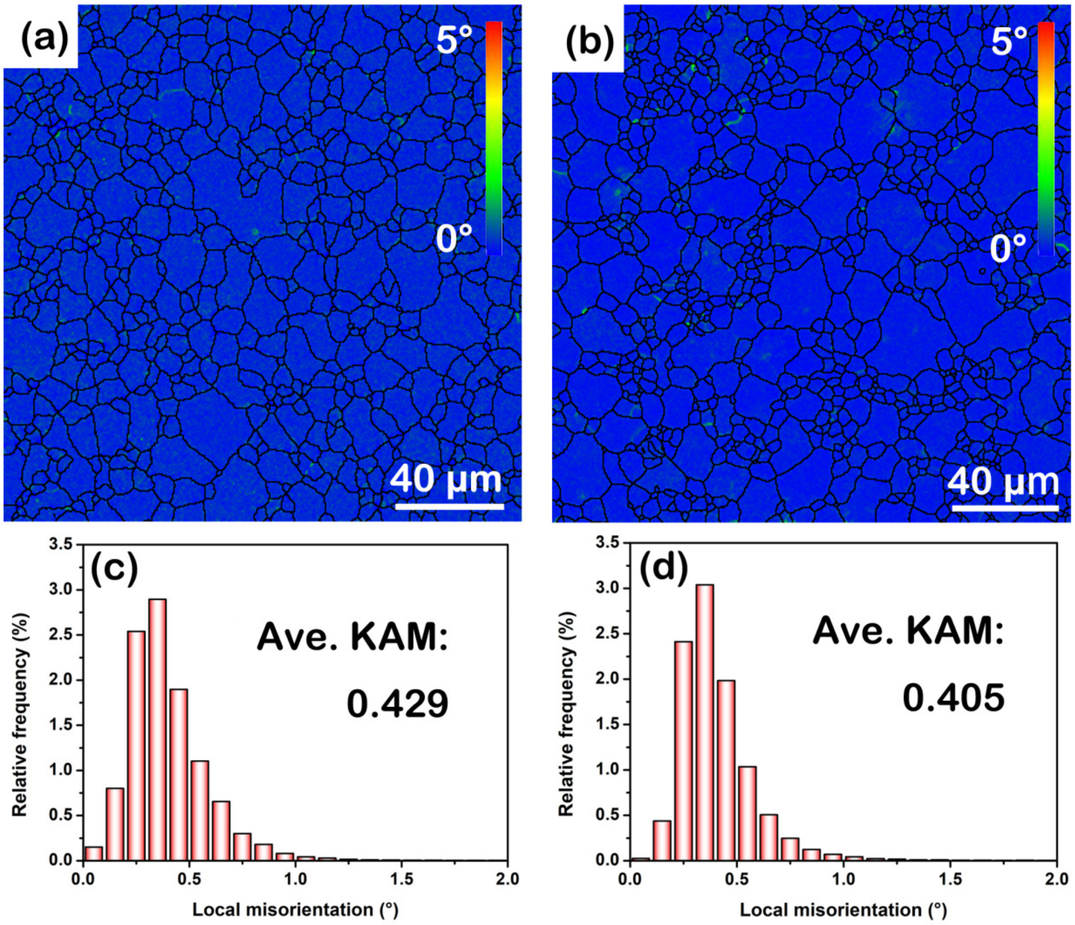

Figure S2. (**a**,**b**) KAM maps; (**c**,**d**) KAM histogram maps of the as-extruded AZ31 and AZB313 alloys, respectively.

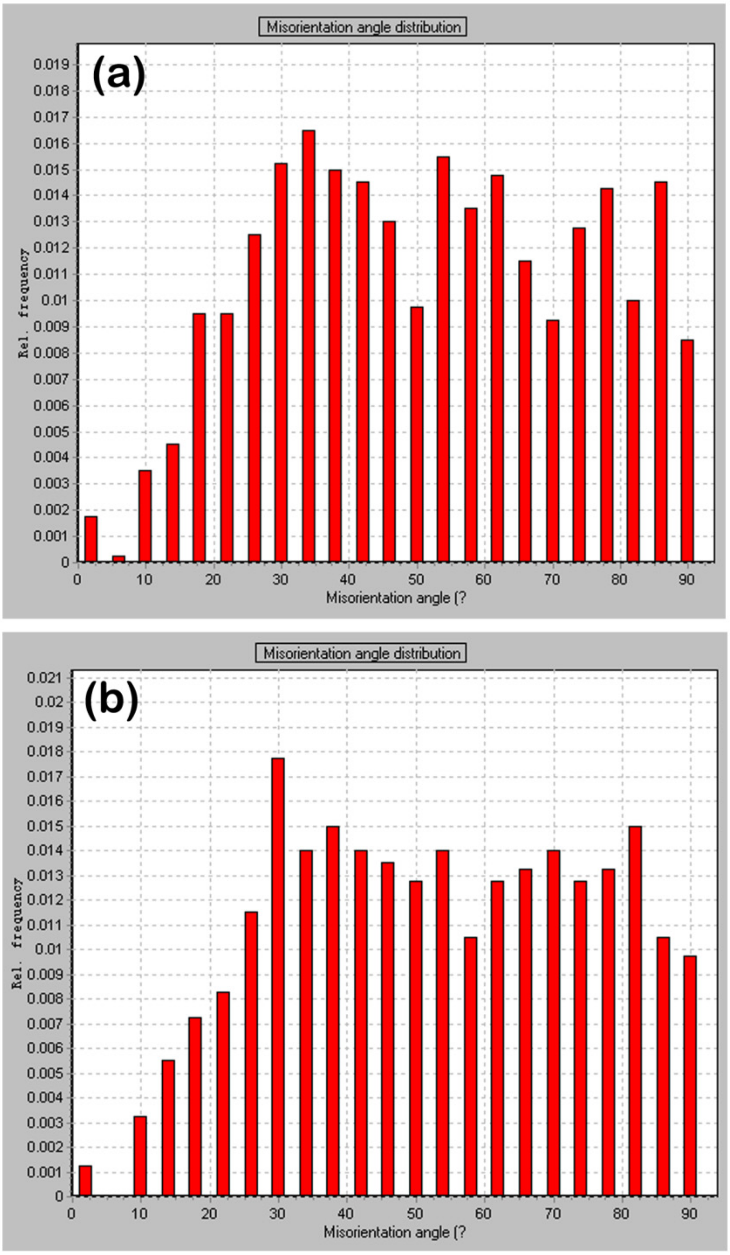

Figure S3. (**a**,**b**) Misorientation angle distribution maps of the as-extruded AZ31 and AZB313 alloys, respectively.
